# Effects of curcumin and metformin on oxidative stress and apoptosis in heart tissue of type 1 diabetic rats

**DOI:** 10.34172/jcvtr.2022.23

**Published:** 2022-06-28

**Authors:** Atefeh Naghdi, Mohammad Taghi Goodarzi, Jamshid Karimi, Mohammad Hashemnia, Iraj Khodadadi

**Affiliations:** ^1^Department of Clinical Biochemistry, School of Medicine, Hamadan University of Medical Sciences, Hamadan, Iran; ^2^Department of Biochemistry, Shahrood Branch, Islamic Azad University, Shahrood, Iran; ^3^Department of Pathobiology, Faculty of Veterinary Medicine, Razi University, Kermanshah, Iran

**Keywords:** Apoptosis, Curcumin, Diabetes Mellitus, Metformin, Oxidative Stress

## Abstract

***Introduction:*** Hyperglycemia enhances oxidative stress and apoptosis and induces damages in heart tissue. Based on antioxidant properties of curcumin and metformin, we hypothesized that these agents may exhibit cardioprotective effects by attenuating oxidative stress and modulating expression of the genes involved in apoptosis in type-1 diabetes.

***Methods:*** Thirty-six male rats were randomly divided into six groups; (N): control; (D): streptozotocin-induced diabetic rats; (D+Cur50) and (D+Cur150): diabetic rats treated with 50 and 150 milligram of curcumin per kilogram of body weight (mg/kg.bw), respectively; (D+Met300) and (D+Met500): diabetic rats received 300 and 500 mg/kg.bw of metformin, respectively. Heart tissues were dissected and gene expression levels of Bax, Bcl-2, and caspase-3 were analyzed. Total anti-oxidant capacity (TAC), total oxidant status (TOS), and malondialdehyde (MDA) level, and activities of catalase (CAT), superoxide dismutase (SOD) and glutathione peroxidase (GPx) were measured.

***Results:*** Enhancement in TOS, OSI, and MDA levels as well as increased in the activity of CAT and reduction in SOD and GPx activities were observed in diabetic group (D) compared with control rats. Treatment of diabetic animals with either curcumin or metformin normalized TOS, OSI, and MDA levels and restored CAT, SOD, and GPx activities. Diabetes caused extensive damages in heart tissue of rats (group D) and increased expression of caspase-3 and Bax genes and enhanced ratio of Bax/Bcl-2 expression compared with controls. Treatment with curcumin or metformin mitigated histopathological changes and dampened apoptosis by normalizing Bax and caspase-3 expression.

***Conclusion:*** Curcumin and metformin modulated diabetes-induced cardiac damage probably by reducing oxidative stress.

## Introduction

 Diabetes mellitus (DM), as one of the most common metabolic disorders, is characterized by hyperglycemia and comes from defects in the insulin secretion by the pancreas and insulin resistance in the end-organ tissues or both.^1^ The prevalence of DM is growing in the world and it is estimated to reach up to 693 million by 2045.^2^ Chronic hyperglycemia results in damage and disrupted functions of multiple organs especially, eyes, kidneys, nervous and cardiovascular systems. Cardiovascular disorders (CVD) remain the most important causes of death and disability in DM-affected patients.^3^ Despite several lines of evidence suggesting CVD risks among diabetic patients, the pathophysiologic mechanisms which link these two diseases are yet to be clearly understood.^2^

 DM-associated CVD includes a range of clinical conditions, such as diabetic cardiomyopathy, coronary heart disease and congestive heart failure, where oxidative stress seems to be involved in pathogenesis of all these conditions.^4^ Hyperglycemia-induced intracellular changes finally lead to generate reactive oxygen species (ROSs) which promote apoptosis process.^5^ Apoptosis is considered as a major player of CVD because it leads to loss of cardiomyocytes. Cardiac regenerative capacity is limited in adult mammalian, thus attempts to prevent losing of cardiomyocytes or stimulating formation of new muscles in damaged heart have received much attention.^6^

 Besides common therapeutic strategies, administration of natural compounds is increasing as major medicinal alternatives, because of their safety, success in the clinical conditions and less side effects. Curcumin is a lipophilic polyphenol compound which is extracted as a major active compound from dried rhizomes of turmeric (*Curcuma longa*).^7,8^ Available evidence suggests that curcumin protects organs against oxidative stress. in addition to the molecular structure-associated antioxidant nature of the curcumin, this compound increases the expression of antioxidant enzymes such as superoxide dismutase (SOD), glutathione peroxidase (GPx) and catalase (CAT) through upregulation of nuclear factor erythroid 2–related factor 2 (Nrf-2).^9,10^ Available evidence also suggests that metformin has antioxidant, anti-inflammatory, and anti-apoptotic effects and might be therapeutically beneficial in improving diseases with inflammatory, apoptotic, and oxidative stress basis in their pathogenesis.^11^

 Recently, protective effect of curcumin on the heart in diabetic patients has attracted considerable attention. Curcumin beneficially modulates oxidative stress and improves metabolic abnormalities in cardiomyocytes. The therapeutic potential of curcumin in the treatment of diabetic cardiomyopathy is probably mediated through several mechanisms such as attenuation of oxidative stress and modulation of Sirt1-Foxo1 and PI3K-Akt pathways^12^ and by regulation of calmodulin-dependent protein kinase II (CaMKII) and peroxisome proliferator-activated receptor gamma (PPAR-γ) expression.^8^

 Since diabetes is associated with apoptosis in cardiomyocytes^12^ and the role of oxidative stress in evoking apoptosis has already been reported,^3^ we hypothesized that curcumin and metformin may ameliorate apoptotic process in cardiomyocytes of type-1 diabetic animal models due to their antioxidant properties. In addition, due to the involvement of caspase-3 enzyme in apoptotic process we postulated that curcumin and/or metformin may modulate cardiomyocyte apoptosis through regulating of caspase-3 gene expression. Therefore, in the present study, beneficial effects of curcumin and metformin were investigated on the oxide-redox status of cardiac tissue by determining total antioxidant capacity (TAC), total oxidant status (TOS), and lipid peroxidation marker malondialdehyde (MDA). In addition, the activities of enzymes involved in cellular antioxidant defense system including CAT, SOD, and GPx were measured and the expression levels of apoptosis-related genes such as Bax, Bcl-2, and caspase-3 in the heart tissue of streptozotocin-induced type 1 diabetic rats were determined.

## Material and Methods

###  Experimental design

 In this case-control study thirty-six 8 weeks old male Wistar rats (150-200 g) were procured from Animal House of Hamadan University of Medical Sciences (Hamadan-Iran). Animals were housed under standard laboratory conditions in a temperature-controlled room under natural light/dark cycle and unlimited access to water and standard chow diet was provided for the rats. All experiments were performed in accordance with the National Institutes of Health Guide for the Care and Use of Laboratory Animals (NIH Publication No.80-23, revised 1996) and the study was approved by the Research Ethics Committee of Hamadan University of Medical Sciences (IR.UMSHA.REC.1395.563).

 After acclimatization of rats with regular chow diet for one week, all animals were randomly divided into six groups (n = 6) as following: (N): normal control group; (D): diabetic untreated group; (D + Cur50): diabetic group treated with 50 milligram of curcumin per kilogram of body weight (mg/kg.bw); (D + Cur150): diabetic group treated with 50 mg/kg.bw of curcumin; (D + Met300): diabetic group treated with 300 mg/kg.bw of metformin; and (D + Met500): diabetic group treated with 500 mg/kg.bw of metformin. Metformin was given to the rats at the concentrations of 300 and 500 mg/kg.bw while rats received curcumin at the 50 and 150 mg/kg.bw concentrations based on previous studies.^13-15^ Metformin and curcumin were purchased from Sigma-Aldrich (Sigma-Aldrich, Ontario-Canada), dissolved and suspended in deionized water, and were orally administrated each day for six weeks (42 days).

###  Induction of diabetes

 After acclimatization of rats with regular chow diet for one week and, streptozotocin (STZ) (Santa Cruz Biotechnology, Heidelberg-Germany) was dissolved in sodium citrate buffer (0.1 M, pH: 4.5) and a dose of 55 mg per kg of body weight was intraperitoneally injected to the rats to induce type 1 diabetes mellitus (T1DM) in diabetic groups (D, D + Cur50, D + Cur150, D + Met300, and D + Met500). Seventy-two hours later, the level of fasting blood sugar (FBS) was measured using a glucometer to confirm induction of diabetes. FBS level higher than 250 mg/dl was considered as diagnostic off point of diabetes.^16^ After induction of diabetes (day zero; D0) treatment of the rats with curcumin or metformin was started and continued for 42 days.^14^ Twenty-four hours after the last treatment, the rats were anesthetized with ketamine–xylazine (50mg/kg ketamine and 12mg/kg xylazine) and sacrificed. Blood samples were collected from jugular vein and sera were separated and stored at -20°C until analysis of biochemical parameters. Heart tissue samples were dissected and used to determine oxide-redox status and to evaluate gene expression levels. Portions of tissue samples were also fixed in 10% neutral-buffered formalin for histopathological evaluation.

###  Analysis of serum biochemical parameters 

 Serum total creatine kinase (CK), creatine kinase MB isoform (CK-MB), lactate dehydrogenase (LDH), and aspartate transaminase (AST) activities were measured using commercially available kits (Parsazmoon, Iran) and a BS-480 Mindray Chemistry Analyzer (Shenzhen Mindray Bio-Medical Electronics Co., Guangdong-China).

###  Determination of oxide-redox status in heart samples

 About 100 mg of heart tissues were rinsed with ice-cold saline, crushed in liquid nitrogen, and homogenized by 1 ml of lysis buffer containing DTT 0.5 mM, HEPES 10 mM, KCl 10 mM, MgCl_2_ 1.5 mM and Triton X100 2%. Homogenates were than centrifuged at 14,000 × g for 20 min (4°C). The supernatants were aliquoted and total protein contents of samples were measured by Bradford assay using bovine serum albumin (BSA) as standard.^17^

 Total antioxidant capacity (TAC) was measured by ferric reducing antioxidant power (FRAP) method.^18^ The principle of FRAP method was based on the reduction of ferric tripyridyl triazine (Fe ^+ 3^-TPTZ) to ferropyridyl triazine (Fe ^+ 2^-TPTZ) by the antioxidant content of the sample. The absorbance of the Fe ^+ 2^-TPTZ complex (blue color) was assayed at 593 nm and the TAC levels were calculated based on a standard curve (FeSO_4_.7H_2_O) and expressed as nmol/mg of protein.

 Total oxidant status (TOS) assay was carried out by ferrous ion oxidation xylenol orange (Fox1) method.^19^ The Fox1 method was based on the oxidation of Fe ^+ 2^ ions to Fe ^+ 2^ in the presence of sample’s oxidizing agents under the acidic medium. The absorbance of Fe ^+ 3^-xylenol orange color complex was read at 546 nm and TOS levels were calculated against H_2_O_2 _as a standard and expressed as µmol/mg of protein.

 Malondialdehyde (MDA), as a lipid peroxidation marker was determined by spectrofluorometric method.^20^ Briefly, the reaction of thiobarbituric acid with MDA led to the formation of a florescence complex with excitation and emission wavelengths at 530 and 550 nm, respectively. Serial dilution of tetraethoxypropane was used as standard and MDA levels were reported as µmol/mg of protein.

###  Determination of catalase activity in heart tissue 

 Catalase (CAT) activity was determined using spectrophotometric Hadwan method^21^ based on the decomposition of H_2_O_2_ by the catalase activity of the samples. Briefly, to 100 µl heart tissue lysate, 1 ml of H_2_O_2 _or deionized water was added (test and control tubes, respectively) while in standard tube 1 ml deionized water was mixed with 100 µl of H_2_O_2_. After incubation of tubes in 37°C for 3 min, enzyme reaction was stopped by 4 ml of ammonium molybdate and absorbances of the tubes were measured at 374 nm. Finally, CAT activity was calculated by the following formula where t was time, S°, S, and M were absorbances of standard, test, and control tubes respectively, Vt was total volume of reagents in test tube, and Vs was volume of supernatant.


$$CAT\;activity=\frac{2.303}{t}\times \left(\log\frac{S^{{}^{\circ}}}{S-M}\right)\times \frac{Vt}{Vs}$$

###  Determination of glutathione peroxidase activity in heart tissue 

 Glutathione peroxidase (GPx) activity in heart tissue samples was determined using Paglia and Valentine method, as previously described.^22^ Briefly, 100 μl NADPH (8 mM), 100 μl reduced glutathione, 20 µl glutathione reductase (30 U/ml) and 20 µl sodium azide (0.12 M) were added to 50 μl of heart lysate. Next, potassium phosphate buffer (50 mM, pH = 7.5) was added to the tubes and mixtures were incubated at 37°C for 30 minute. Then, 100 `μl H_2_O_2 _was added and the reductions in the absorbance at 340 nm were measured. Finally, GPx activities were calculated as μmol of NADPH used to reduce glutathione.

###  Determination of superoxide dismutase activity in heart tissue 

 Superoxide dismutase (SOD) activity was measured by the Marklund method^23^ in which one unit of enzyme activity was equal to the quantity of enzyme required to inhibit 50% of pyrogallol oxidation rate. Briefly, 2 ml Tris-HCl (50 mM, pH = 8.2) and 20 µl pyrogallol solution (10 mM, pH = 7.4) were added to 30 µl heart lysate and absorbances of test and control tubes were measured at 420 nm. Finally, the results of enzyme activity were calculated based on the inhibition of pyrogallol autoxidation percent [(∆A test/∆A control) × 100]/50% and expressed as U/mg of protein.

###  Determination the expression levels of apoptosis-related genes by RT-qPCR

 Gene expression levels of Bax, Bcl-2, and caspase-3 in heart tissue samples were determined by RT-qPCR on a Roche Light Cycler^®^ 96 System (Roche Life Science, Sandhofer-Germany) and qPCR master mix (QuantiFast SYBR Green PCR Kit). Briefly, total RNA was isolated using RNX-plus reagent (Cinnagen, Iran), according to the manufacturer’s protocol`. The quantity and quality of the isolated RNA were evaluated by NanoDrop^TM^ (Thermo Fisher Scientific-USA) and 1% agarose gel electrophoresis, respectively. cDNA was synthesized by reverse transcription of 500 ng of total RNA using PrimeScript first Standard cDNA Synthesis Kit (Takara Biotechnology, Japan). Gene-specific primers (Table 1) were designed using AlleleID^®^6 software (Premier Biosoft Corporation, USA). Online primer blast software (https://www.ncbi.nlm.nih.gov/tools/primer-blast/) was used to confirm specificities of designed primers to the corresponding genes. β-actin was considered as housekeeping gene and relative mRNA expression levels (fold change) were calculated by 2 ^-∆∆Ct^formula based on Livak method^24^ compared with the expression of β-actin.

**Table 1:** Sequences and characteristics of primers used in the study.

**Gene name**	**Accession number**	**Sequences**	**Tm°C**	**Product size (bp)**
β-Actin	NM_031144.3	F: 5'-ATCAGCAAGCAGGAGTACGAT-3'	56	94
		R: 5'-AAAGGGTGTAAAACGCAGCTC-3'		
Bcl-2	NM_016993.1	F: 5′-TGTGGATGACTGAGTACCTGAACC-3′	52	122
		R: 5′-CAGCCAGGAGAAATCAAACAGAGG-3′		
Bax	NM_017059.2	F: 5'-GCAGAGGATGATTGCTGATGTGG-3'	52	204
		R: 5'-AGGAAGTCCAGTGTCCAGCCCAT-3'		
Caspase-3	NM_012922.9	F: 5'-ACAACAACGAAACCTCCGTG-3'	56	192
		R: 5'-CATTGCGAGCTGACATTCCA-3'		

Gene sequences were obtained from online Gene Bank database (https://www.ncbi.nlm.nih.gov/genbank/). Gene-specific primers were designed using AlleleID^®^6 software (Premier Biosoft Corporation, USA). Online primer blast software (https://www.ncbi.nlm.nih.gov/tools/primer-blast/) was used to confirm specificities of designed primers to the corresponding genes.

###  Histopathological evaluation

 For histopathological studies, heart tissue samples were fixed in 10% neutral-buffered formalin, processed, and embedded in paraffin. Tissue samples were then sectioned into 5 μm thickness slides and stained with hematoxylin and eosin (H&E). Finally, the slides were examined by a pathologist using a routine light microscope and characteristics of cardiomyocyte structure including uniformity, shape and location of nuclei and intercalated discs of plasma membrane were evaluated. Presence of hypertrophic myofibers, vacuolation and congestion of cytoplasm, and deformation of nuclei were also investigated.

###  Statistical analysis

 Statistical analysis was carried out using the SPSS 16 (SPSS Inc., Chicago-USA). One-way ANOVA followed by post hoc Tukey test was used for comparison between groups. Data were presented as mean ± SD and *P *values less than 0.05 (*P* < 0.05) was considered as statistical significance.

## Results

###  Effects of curcumin and metformin on serum biochemical parameters 

 As shown in Table 2, fasting blood sugar was significantly higher in all treated or untreated diabetic rats compared with control rats at the end of study (FBS D42). Although treatment with 150 mg/kg.bw of curcumin slightly lowered FBS compared with untreated diabetic animals (group D), the results confirmed that neither of low or high doses of curcumin or metformin show notable hypoglycemic property in type 1 diabetes. AST significantly (*P* < 0.001) increased in serum to a mean of 296.66 U/L after induction of diabetes (D group) but treatment of the rats with curcumin (*P*= 0.006 and *9* = 0.009 for low and high doses) or metformin (*P* = 0.004 and *P* = 0.026 for low and high doses) successfully lowered AST level compared with group D. At the end of experiment (day 42) no significant difference was observed in AST level between treated rats and control animals. Despite of significant increase in the level of LDH in diabetic group (D) compared with the control group, treatment with curcumin and metformin almost completely restored LDH level to normal level (*P*< 0.05). As shown in Table 2, the activity of serum creatine kinase (CK) and its CK-MB isoform did not significantly differ between control and diabetic groups (N and D, respectively), however treated rats (D + Cur50, D + Cur150, D + Met300, and D + Met500) showed almost 50% lower CK and CK-MB enzyme activities compared with both control and untreated diabetic rats in D group. Since the pattern of alteration in total CK activity was similar to that of CK-MB, the CK-MB/CK ratio was calculated for further evaluation of creatin kinase activity in diabetes. Diabetic rats (group D) showed higher CK-MB/CK ratio than control group (3.72 ± 0.15 vs 3.27 ± 0.12, *P* < 0.01) while treatment with both doses of curcumin in groups D + Cur50 and D + Cur150, resulted in significant reduction in CK-MB/CK ratio compared with untreated diabetic rats (*P*= 0.026 and *P =*0.015, respectively) and completely restored the ratio level to that of control rats. Treatment with metformin showed relatively mild effect and despite to reducing CK-MB/CK ratio to the normal range, it did not significantly differ from that of diabetic rats (group D).

**Table 2:** Effects of curcumin or metformin on biochemical parameters

**Groups**	**FBS D42 (mg/dl)**	**AST (U/L)**	**LDH (U/L)**	**CK (U/L)**	**CK-MB (U/L)**	**CK-MB/CK**
N	92.33 ± 7.52	163.33 ± 15.05	1268.33 ± 177.81	1511.66 ± 686.18	493.33 ± 215.19	3.27 ± 0.12
DM	586.67 ± 16.14^a^	296.66 ± 52.40^a^	1966.66 ± 186.19^a^	1218.33 ± 431.15	403.33 ± 138.94	3.72 ± 0.15^a^
D + Cur50	570.50 ± 21.03^a^	208 ± 27.75^b^	1356.66 ± 520.48^b^	581.66 ± 239.28^a^	228.57 ± 74.41^a^	3.3 ± 0.26^b^
D + Cur150	541.60 ± 27.72^a,b^	212 ± 25.88^b^	1340 ± 380.98^b^	650 ± 195.32^a^	244 ± 72.66^a^	3.24 ± 0.34^b^
D + Met300	590.50 ± 12.72^a,c^	212.85 ± 24.30^b^	1362.85 ± 252.50^b^	630 ± 265.20^a^	228.57 ± 81.94^a^	3.35 ± 0.22
D + Met500	588.60 ± 11.97^a,c^	222 ± 59.33^b^	1396 ± 309.56	610 ± 306.35^a^	228 ± 102.057^a^	3.43 ± 0.11

N: Normal group; DM: Diabetic group, D + Cur50: Diabetic group treated with 50 mg/Kg curcumin, D + Cur150: Diabetic group treated with 150 mg/Kg curcumin, D + Met300: Diabetic group treated with 300 mg/Kg metformin); D + Met500: Diabetic group treated with 500 mg/Kg metformin. FBS: Fasting blood sugar; AST: Aspartate transaminase; LDH: Lactate dehydrogenase; CK: Total creatine kinase; CK-MB: creatine kinase MB. Data is represented as mean ± SD and *P* < 0.05 is considered as statistically significance difference. In each column “a” represents significant difference with normal group (N), “b” indicates significant difference with diabetic group (DM), and “c” shows significance difference with D + Cur150.

###  Antioxidant properties of curcumin and metformin in heart tissue

 Heart level of TAC showed no significant difference between studied groups (Table 3). On the other hand, TOS level was significantly higher in group D than the control group (*P*< 0.001). Although TOS level in curcumin- and metformin-treated diabetic rats were relatively lower compared with untreated diabetic rats (group D), this difference was statistically significance only in D + Cur50 and D + Cur150 groups (*P*= 0.015 and *P*= 0.007, respectively). As shown in Table 3, determination of oxidative stress index (OSI) as a ratio of TOS to TAC showed that induction of diabetes markedly increased OSI in D group (*P*= 0.013) but treatment of the diabetic animals with both low and high doses of curcumin or metformin successfully lowered OSI compared with un-treated diabetic rats (group D) and normalized OSI to the level observed in control group. Likewise, lipid peroxidation marker (MDA) which was increased over 20% in D group (*P*= 0.005) was markedly reduced in all D + Cur50, D + Cur150, D + Met300, and D + Met500 treated rats compared to group D (*P*= 0.008, *P*= 0.007, *P* = 0.001, and *P* = 0.029 respectively) and completely restored to the normal level. Interestingly, no difference was observed in efficiency of curcumin and metformin in lowering lipid peroxidation level, as indicated in Table 3.

**Table 3:** Effects of curcumin and metformin on antioxidant parameters

**Groups**	**TAC ** **(nmol/mg pr)**	**TOS** **(nmol/mg pro)**	**OSI** **(arbitrary)**	**MDA ** **(μmol/mg pr)**
N	0.52 ± 0.13	0.82 ± 0.05	1.62 ± 0.30	0.38 ± 0.02
DM	0.53 ± 0.07	1.03 ± 0.05^a^	2.09 ± 0.31^a^	0.46 ± 0.03^a^
D + Cur50	0.53 ± 0.06	0.89 ± 0.10^b^	1.67 ± 0.16^b^	0.39 ± 0.03^b^
D + Cur150	0.55 ± 0.12	0.87 ± 0.06^b^	1.60 ± 0.19^b^	0.38 ± 0.02^b^
D + Met300	0.52 ± 0.07	0.93 ± 0.03	1.66 ± 0.08^b^	0.38 ± 0.05^b^
D + Met500	0.54 ± 0.05	0.92 ± 0.09	1.67 ± 0.17	0.39 ± 0.02^b^

N: Normal group; DM: Diabetic group, D + Cur50: Diabetic group treated with 50 mg/Kg curcumin, D + Cur150: Diabetic group treated with 150 mg/Kg curcumin, D + Met300: Diabetic group treated with 300 mg/Kg metformin); D + Met500: Diabetic group treated with 500 mg/Kg metformin. TAC: Total antioxidant capacity; TOS: Total oxidant status; MDA: Malondialdehyde; OSI: oxidative stress index. Data is represented as mean ± SD and *P* < 0.05 is considered as statistically significance difference. In each column “a” represents significant difference with normal group (N) and “b” indicates significant difference with diabetic group (DM).

###  Curcumin or metformin favorably restored activities of antioxidant enzymes in heart 

 Determination of activities of enzymes involved in antioxidant defense system showed that (Table 4) although induction of diabetes (D group) significantly increased CAT activity (*P*= 0.006), treatment with curcumin and metformin prevented alteration in CAT activity where no significant difference was observed in the activity of CAT compared with normal group. Unlike catalase, both SOD and GPx decreased by the induction of diabetes (*P*= 0.001 for both enzymes), as observed in group D. However, reduction in the activity of both enzymes was completely prevented by the treatment of the rats using either low or high doses of curcumin and metformin in all treated animals (D + Cur50, D + Cur150, D + Met300, and D + Met500), as shown in Table 4. Interestingly, treatment with curcumin (both low and high doses) were more effective than metformin in preventing declines in SOD and GPx activities caused by the induction of diabetes in D + Cur50 and D + Cur150 groups.

**Table 4:** Effects of curcumin and metformin on the activity of enzymes involved in antioxidant system

**Groups**	**CAT (U/mg pr)**	**SOD (U/mg pr)**	**GPx (U/mg pr)**
N	19.34 ± 0.94	37.64 ± 0.82	26.19 ± 1.00
DM	22.75 ± 1.72^a^	35.74 ± 0.90^a^	24.66 ± 0.49^a^
D + Cur50	21.12 ± 1.42	37.55 ± 0.75^b^	25.78 ± 0.15^b^
D + Cur150	21.57 ± 1.31	37.52 ± 0.78^b^	25.99 ± 0.10^b^
D + Met300	20.78 ± 1.03	36.68 ± 0.31	25.35 ± 0.42
D + Met500	21.25 ± 1.96	36.92 ± 0.26	25.35 ± 0.58

N: Normal group; DM: Diabetic group, D + Cur50: Diabetic group treated with 50 mg/Kg curcumin, D + Cur150: Diabetic group treated with 150 mg/Kg curcumin, D + Met300: Diabetic group treated with 300 mg/Kg metformin); D + Met500: Diabetic group treated with 500 mg/Kg metformin. CAT: Catalase; SOD: Superoxide dismutase; GPx: Glutathione peroxidases. Data is represented as mean ± SD and *P* < 0.05 is considered as statistically significance difference. In each column “a” represents significant difference with normal group (N) and “b” indicates significant difference with diabetic group (DM).

###  Effects of curcumin or metformin on gene expression of Bax, Bcl-2, and Caspase-3 in heart

**Figure 1:**
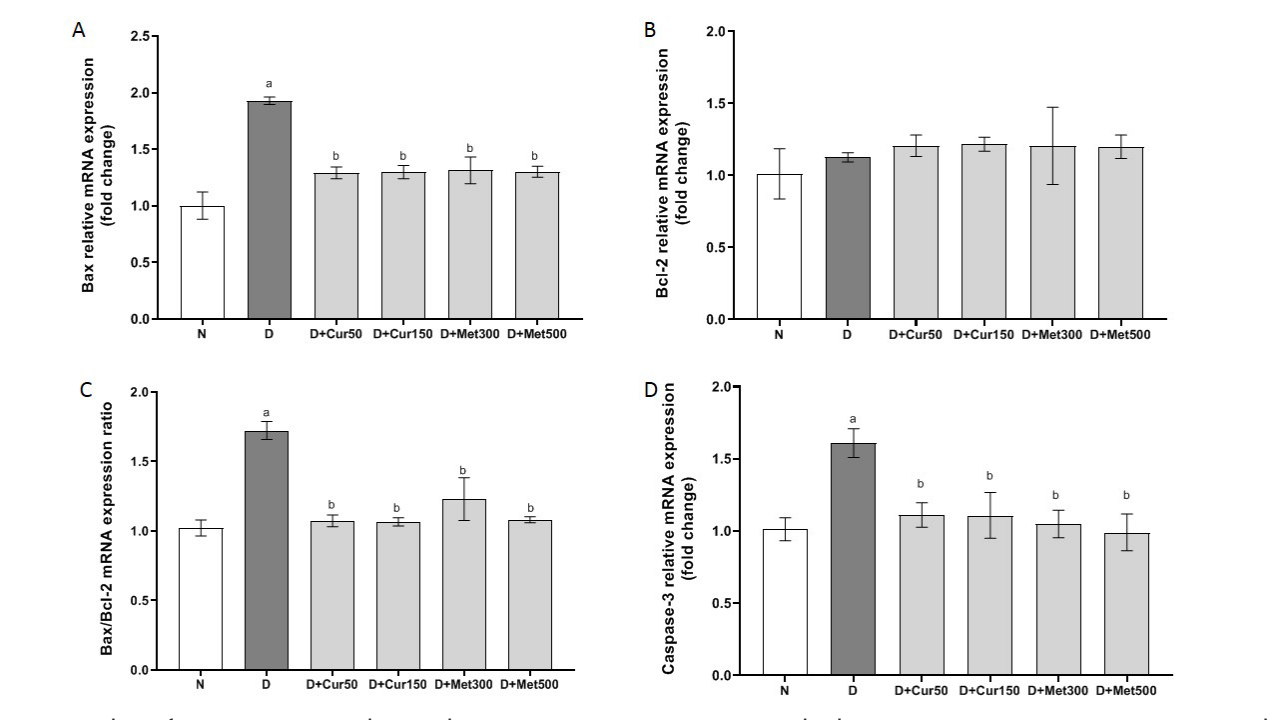
Effects of curcumin and metformin on Bax, Bcl-2, and Caspase-3 gene expression in the heart tissue: (A) Bax expression, (B) Bcl-2 expression, (C) Caspase-3 expression and (D) Bax-/Bcl-2 expression ratio, as determined by RT-qPCT. N: normal group; D: diabetic group D+Cur50: diabetic group treated with curcumin (50 mg/kg), D+Cur150: diabetic group treated with curcumin (150 mg/kg), D+Met300: diabetic group treated with metformin (300 mg/kg); D+Met500: diabetic group treated with metformin (500 mg/kg). Data is represented as mean ± SD. In each figure «a» represents significant difference (p<0.001) with normal group (N) and «b» indicates significant difference (p<0.001) with diabetic group (D)

 The gene expression analysis revealed that expression of Bax was almost doubled (*P*< 0.001) by the induction of diabetes in the rats (D group), as shown in Figure 1A, but treatment of animals with low or high doses of curcumin or metformin significantly suppressed evaluation of Bax gene expression in treated rats in D + Cur50, D + Cur150, D + Met300, and D + Met500 groups (*P*< 0.001 for all). Unlike the increment in the expression of proapoptotic gene (Bax) by the induction of diabetes in D group, the expression of antiapoptotic Bcl-2 gene neither altered by inducing of diabetes (in group D) nor by the treatment of the diabetic rats with curcumin or metformin (Figure 1B). However, the ratio of Bax/Bcl-2 gene expressions which determines cell susceptibility to apoptosis showed higher susceptibility of heart tissues to apoptosis in diabetic rats from group D compared with control rats (*P*< 0.001) whereas all curcumin- or metformin-treated animals (D + Cur50, D + Cur150, D + Met300, and D + Met500) showed almost statistically (*P*< 0.001) similar lower apoptotic susceptibility (Figure 1C). Antiapoptotic properties of curcumin and metformin were further confirmed by determination of caspase-3 gene expression which showed over 50% elevation (*P*< 0.001) in gene expression in diabetic animals (D group) while none of curcumin- or metformin-treated rats showed significant changes in caspase-3 gene expression level (Figure 1D).

###  Curcumin and metformin prevented diabetes-induced tissue damages

**Figure 2:**
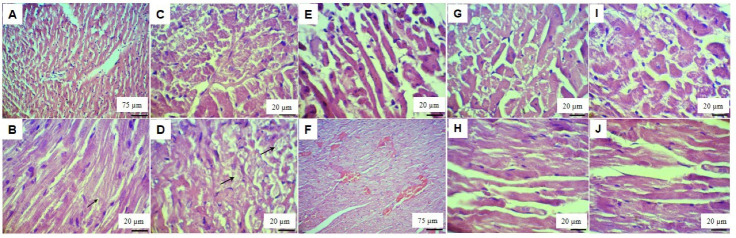
Curcumin and metformin protected cardiac tissue from diabetes-induced damages, as shown by histopathological examinations. In control rats, (A) histopathology of normal heart tissue with normal features of myocardial cells (H&E; Bar=75 µm) and Normal myocardial cells with normal nuclei, longitudinal striation of fibers and intercalated discs, as indicated with arrows (B) were observed (H&E; Bar=20 µm). In contrast, in untreated diabetic rats (group D), hypertrophic myofibers with disarrayed pattern (H&E; Bar=20 µm) were observed (C) and cardiomyocytes degeneration with vacuolated cytoplasm was detected (D), as shown by arrows (H&E; Bar=20 µm). In addition, degenerated cardiomyocytes with pyknotic nuclei and eosinophilic cytoplasm (H&E; Bar=20 µm) as shown in (E) and congested blood vessels (F) were also observed in diabetic rats (H&E; Bar=75 µm). After treatment of the diabetic rats with low dose of curcumin (group D+Cur50) a few degenerated myocardial cells with vacuolated cytoplasm and mild hypertrophy was observed (G) while higher dose of curcumin (group D+Cur150) resulted in mild myocytes hypertrophy with a minimal degree of cytoplasmic vacuolation (H). Similarly, in those diabetic rats received lower dose of metformin (D+Met300), degenerated myocardial cells with vacuolated cytoplasm (H&E; Bar=20 µm) were seen (I) but treatment of the rats with 500 mg/kg of metformin (D+500Met) mostly prevented diabetes-induced damages (J) and a few myocardial cells with mild hypertrophy (H&E; Bar=20 µm) was detected in heart tissue.

 Histopathological examination of the H&E-stained heart sections of control rats revealed normal structure. The cardiomyocytes were uniform with single, oval and centrally located nuclei and the plasma membrane and intercalated discs were intact (Figure 2) whereas induction of diabetes led to extensive structural changes in heart tissue and the presence of hypertrophic myofibers with disarrayed pattern, a mild distortion of intercalated discs, degenerated cardiomyocytes with vacuolated cytoplasm and moderate congestion were the main histopathologic findings. Moreover, nuclei of the cardiomyocytes showed deformation in sizes and shapes (Figure 2).

 Treatment with low doses of curcumin (D + Cur50) and metformin (D + Met300), though could not completely alleviate the lesions, showed milder degenerative changes such as nuclear alteration, vacuolated cytoplasm and congestion, when compared to untreated diabetic rats (Figure 2). Heart sections of diabetic rats treated with high doses of curcumin (D + Cur150) and metformin (D + Met500) showed no pathological changes and were comparable to those of normal rats except the presence of small cytoplasmic vacuoles and mild myocytes hypertrophy (Figure 2).

## Discussion

 The major mechanisms involved in the pathogenesis of diabetes-induced heart disorders are not yet clearly understood. It is believed that oxidative stress and inflammation resulted from diabetes play critical role in linking diabetes and cardiac disorders through induction of apoptosis.^25^ In diabetes, hyperglycemia triggers intracellular changes leading to the generation of ROS which in turn promotes cellular apoptosis and loss of cardiomyocytes in CVD.^5,26^ Nowadays, besides common therapeutic strategies using natural compounds as alternative or adjuvant therapeutic agents has received much attention due to their beneficial therapeutic potency, lake of side effects and safety. Available evidence suggests that curcumin which is extracted as major lipophilic polyphenol from turmeric^7,8^ exhibits antioxidant potential^27^ and upregulates the expression of antioxidant enzymes of cellular defense system.^9,10^ Similarly, antioxidant, anti-inflammatory, and anti-apoptotic properties have also been reported for metformin in some experimental conditions.^28,29^ Thus, based on the antioxidant effects of curcumin and metformin, we hypothesized that these compounds may inhibit oxidative stress-induced apoptosis in heart tissue of diabetic rats. Therefore, in the present study beneficial effects of curcumin and metformin on heart tissue were investigated by determining TAC, TOS, OSI, and MDA levels and measuring the activities of enzymes involved in cellular antioxidant defense system including CAT, SOD, and GPx. In addition, the expression levels of apoptosis-related genes such as Bax, Bcl-2, and caspase-3 were determined by RT-qPCR.

 We showed that induction of diabetes in D group led to the significant increase in serum AST and LDH levels compared with the control groups which is in line with previous reports.^30,31^

 Interestingly, treatment with both low and high doses of curcumin or metformin significantly prevented alteration of AST and LDH and restored them to the normal range. It is postulated that increased production of ROS, as the consequences of hyperglycemia, leads to damage of the cardiomyocytes cell membrane through reaction with lipids, proteins and other cellular constituents, and ultimately results in the releasing of AST and LDH from damaged cardiac cells into the circulation.^5,32^ The beneficial effects of curcumin and metformin in restoring AST and LDH activities, as observed in this study, are probably attributed to the antioxidant nature of curcumin and metformin,^28,29^ which protected cardiomyocytes from oxidative damages.

 Although in the present study none of total CK and its cardiac isoform (CK-MB) increased in diabetic rats (D group), as expected based on the previous reports in the literature,^30,33^ the ratio of CK-MB to total CK (defined as CK-MB/CK) was significantly increased by the induction of diabetes in the rats. Treatment of the rats with either curcumin or metformin normalized the CK-MB/CK ratio to that of control animals but curcumin was found more effective than metformin in restoring CK-MB/CK ratio. Unexpectedly, despite normalizing of CK-MB/CK ratio treatment of diabetic rats with curcumin or metformin lowered total CK and CK-MB levels to nearly 50% of their normal levels. At the moment underlying molecular mechanism of action of metformin or polyphenolic curcumin in greatly lowering CK and CK-MB activities is not clear and further studies are required to show whether reduction in enzyme activities is due to the downregulation at the transcriptional level, suppressing translational stage, or inhibition of post-translational modification processes. Nevertheless, it is supposed that antioxidant properties of these compounds may play critical roles in their wide-range and multifaced cardioprotective potentials.

 In the present study although TAC level did not alter by the induction of diabetes or even by the treatment of diabetic rats with curcumin or metformin, significant enhancements were observed in TOS, OSI, and MDA levels in rats from diabetic group (D). In contrast, treatment of diabetic rats (D + 50Cur, D + 150Cur, D + 300Met, and D + 500Met groups) completely returned these parameters of oxidative stress to their normal ranges, being curcumin more effective than metformin. Similar to our observation, a previously published report has stated increasing in TOS level in heart tissue without any significant changes in TAC level in diabetic rats.^34^ Increased TOS level is an expected finding in diabetes mellitus because diabetes-induced hyperglycemia eventually leads to increased production of ROS^5^ and ROS itself constitute a major part of TOS.^35^ Taş et al also have shown that diabetes resulted in increased heart MDA level^36^ through peroxidation of lipids^37^ which occurs as a consequence of diabetes-induced enhancement in reactive oxygen species. Available evidence indicated that apart from the antioxidant properties of curcumin which is related to its chemical nature,^9^ curcumin may prevent increasing of ROS through reducing of electron transfer chain and activities of enzymes which produce ROS.^25,38^ Metformin may also decrease ROS production through inhibition of mitochondrial respiratory complex-I and increasing of pentose phosphate pathway activity.^39^

 Surprisingly, results of antioxidant enzymes activities were contradictory in the present study. In line with results of study by Mollazadeh et al,^30^ we showed that diabetic rats had significantly reduced GPx and SOD activities in the heart, whereas showed markedly increased CAT activity compared with control rats. Although this discrepancy in CAT, SOD, and GPx activities can be explained by the fact that Nrf-2 protein may slightly increases in the heart tissue of two-months old diabetic rats where as a transcription factor upregulates expression of genes involved in antioxidant defense system,^40,41^ and therefore, the increased CAT activity in our study might be occurred as a compensatory response against hyperglycemia-induced H_2_O_2_ production.^42^ However, further studies are required to deeply investigate contradictory responses of CAT, SOD, and GPx in diabetes since upregulation of Nrf-2 expression in acute hyperglycemia as well as its downregulation in chronic hyperglycemia are both reported.^40,41^ Treatment with curcumin and metformin completely prevented increasing of CAT activity in the treated groups. Given that increased CAT activity in diabetic rats (D group) is a compensatory response against diabetes-induced oxidative stress,^42^ so treatment with curcumin and metformin probably modulated this response through their antioxidant capacities. Upregulation of Nrf-2 by curcumin and metformin has previously been observed,^9,43^ thus Nrf-2-dependent increase in GPx and SOD transcription (and activity) might be a cause of increased SOD and GPx observed in treated diabetic rats (D + 50Cur, D + 150Cur, D + 300Met, and D + 500Met).

 It has previously been known that diabetes induces apoptosis and cardiomyocyte damage through increased production of ROS.^5,6,26^ Here, we showed that induction of diabetes in DM group up-regulated expression of caspase-3 and Bax gene expression and increased ratio of Bax/Bcl-2. Similar observations confirming of increased expression of caspase-3 and Bax genes and enhanced Bax/Bcl-2 ratio have previously been reported in diabetes.^44^ It is supposed that increased ROS levels in hyperglycemic conditions reduces AKT activation through reduction of its phosphorylation. AKT inhibits transcription factor FOXO3 which participates in the expression of pro-apoptotic genes such as Bax, thus decreased phosphorylation (and activity) of AKT abolishes inhibitory effects on FOXO3 and induces the expression of Bax and increases the chance of formation Bax-Bax homodimers which is in favor of progressing the apoptosis process.^45^ Therefore, the effects of curcumin and metformin in suppressing Bax gene expression might be explained by the fact that curcumin induces phosphorylation and activation of AKT through pro-survival kinase such as phosphoinositide 3-kinase^12^ and metformin reduces pro-apoptotic genes through down-regulation of FOXO3.^39^

 Induction of diabetes resulted in severe damage in heart tissue on diabetic rats in D group. Hypertrophic myofibers with disarrayed pattern, cardiomyocytes degeneration with vacuolated cytoplasm, and congested blood vessels were observed in histopathological evaluation of heart tissue in diabetic rats which are in line with previous studies.^46,47^ Increased ROS level directly or indirectly activates some signal transduction pathways which foster the expression or augment activation of the hypertrophic factors such as beta-myosin heavy chain.^48^ Cardiomyocyte degeneration also may be occurred through autophagy as process which can be mediated through ROS.^49^ In the other hand, histopathological evaluation indicated the presence of pyknotic nuclei in the heart tissue of diabetic rats, which are considered as a small condensed nuclei of apoptotic cells.^50^ Treatment with curcumin and metformin especially in high doses prevented progressing of diabetes-induced tissue damages through their antioxidant and anti-apoptotic effects. Therefore, it can be mentioned that the findings of histopathological evaluation confirmed the results of gene expression and evaluation of oxidative stress in this present study.

 Comparison of beneficial effects of low (50mg/kg) and high (150mg/kg) doses of curcumin showed that there was no statistically significant difference between these two doses in any of parameters measured in the present study including FBS, AST, LDH, CK, CK-MB, CK-MB/CK, TAC, TOS, OSI, MDA, CAT, SOD, GPx, and the expression of Bax, Bcl-2, Bax/Bcl-2, and caspase-3 genes. Similarly, no difference was observed in these parameters between the effects of low (300mg/kg) and high (500mg/kg) doses of metformin. Therefore, it is postulated that wider range of concentrations (higher doses) of curcumin and/or metformin might be needed to see different impacts. However, the probable toxic effects of consuming higher doses of these agents should be taken into consideration in long term treatments.

 Moreover, the effects of curcumin and metformin on dependent variables did not differ between groups in exception to the FBS level, where curcumin was significantly effective than metformin. Although no significant difference was observed between curcumin and metformin in AST, LDH, CK, CK-MB, CK-MB/CK, TAC, TOS, OSI, MDA, CAT, SOD, GPx, and the expression of Bax, Bcl-2, Bax/Bcl-2, and caspase-3 genes, the mean values for almost all parameters in curcumin-treated animals were relatively closer than those of metformin to the normal values determined in control rats.

 Collectively, it can be suggested that the favorable effects of curcumin and metformin in ameliorating oxidative stress and attenuating diabetes-induced damages in cardiomyocytes, as observed here, were more possibly mediated through their antioxidant properties and not because of their hypoglycemic potency, particularly for metformin. These results may be considered as our incipient understanding about effectiveness of curcumin in ameliorating diabetes-induced oxidative stress and apoptosis in the heart tissue since a deeper investigation of the cardioprotective mechanisms of curcumin and/or metformin requires measurement of ROS, evaluation of apoptosis using TUNEL assay or flow cytometry, measurement of caspase-3 enzyme activity, investigating synergistic effects of combined treatments (curcumin + metformin), and determination of Bax and Bcl-2 at the protein level.

## Conclusion

 Curcumin and metformin exhibit strong antioxidant properties and therefore might be beneficial in ameliorating diabetes-induced oxidative stress and modulating diabetes-induced cardiac damage in the heart tissue.

## Acknowledgments

 The authors would like to thank Hamadan University of Medical Sciences for financial support (IR.UMSHA.9512037389).

## Funding

 This study did not receive any specific grant from funding agencies in the public, commercial, or not-for-profit sectors.

## Competing of interests

 The authors have declared no conflicts of interests.

## Ethical approval

 The study was approved by the Research Ethics Committee of Hamadan University of Medical Sciences (IR.UMSHA.REC.1395.563).
